# Bilateral Choroidal Metastases as Presentation of Dissemination of Cutaneous Malignant Melanoma

**DOI:** 10.1155/2012/486167

**Published:** 2012-10-22

**Authors:** S. Fernandez-Perez, O. Ruiz-Moreno, V. Pueyo, G. de la Mata, L. Pablo

**Affiliations:** Ophthalmology Department, Miguel Servet University Hospital, Paseo Isabel la Católica 1-3, 50009 Zaragoza, Spain

## Abstract

*Case Report*. A 47-year-old man presented with blurred vision in the right eye. Ophthalmoscopic examination showed several placoid, pigmented lesions in the posterior pole and midperiphery of the retina of both eyes. *Results*. Patient referred a cutaneous malignant melanoma on the back skin removed 6 years ago. A systemic workup revealed multiple metastases in liver and spleen. After an exhaustive study we concluded that it was a dissemination of a cutaneous malignant melanoma with bilateral choroidal metastases, liver and spleen metastases. The patient obtained clinical ocular improvement after palliative chemotherapy, although he died in the following months. Pathological examination of the lesions confirmed the diagnosis of choroidal metastases from a malignant cutaneous melanoma. *Conclusions*. Monitoring patients who have had cutaneous malignant melanoma is very important, since melanoma metastases may occur even many years after the diagnosis of the primary tumor. Choroidal metastases from cutaneous melanoma are uncommon but we should be aware because their appearance worsens prognosis.

## 1. Introduction

Metastatic tumors are the most common intraocular malignancy, representing the choroid the most common site of intraocular metastases due to its high vascularity. Its clinical presentation is most usual with painless loss of vision and there is a previous history of malignancy in 66% of cases, being more frequent breast cancer in women and lung cancer in men [1].

Dissemination of cutaneous malignant melanoma to the choroid is a rare entity that it worsens prognosis of patients.

We report a patient with bilateral choroidal metastases as the first sign of dissemination of cutaneous malignant melanoma.

## 2. Case Report

A 47-year-old Caucasian male presented to our emergency department with rapid-onset blurred vision in his right eye (RE).

**Figure 1:**
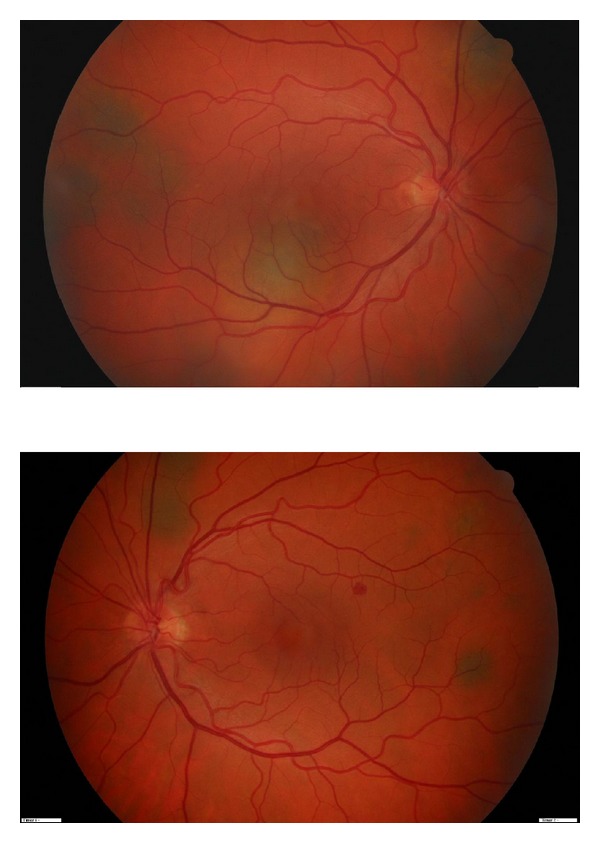
Upper image: funduscopic image of right eye with several placoid pigmented choroidal lesions located in the posterior pole and midperiphery of the retina and serous macular detachment. Lower image: funduscopic image of the left eye with various pigmented choroidal lesions.

**Figure 2:**
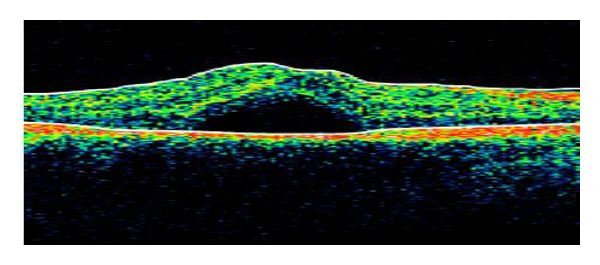
OCT image of the macular serous detachment in the right eye at the time of diagnosis.

The best-corrected visual acuity was 20/64 in the RE and 20/20 in the left eye (LE). Slitlamp examination of anterior chamber was normal. Ophthalmoscopic examination revealed multiple pigmented placoid lesions located in the posterior pole and midperiphery of the retina of both eyes and a serous macular detachment in RE (Figure 1). Optical coherence tomography (OCT) confirmed the macular neurosensory detachment in the RE (Figure 2). Fundus fluorescein angiography showed hypofluorescence during the arterial phase and progressive hyperfluorescence of the lesions during the subsequent phases in both eyes and a complete filling of the neurosensory retinal detachment in the late phases in the RE.

After conducting an exhaustive medical history, the patient referred a cutaneous malignant melanoma in the upper-right side of the back skin removed 6 years ago. The pathological report showed the existence of a superficial spreading cutaneous melanoma 9 × 7 mm, Clark level 3 and Breslow thickness 0.71 mm.

Since clinical suspicion of choroidal metastases, we referred the patient to the Oncology Department for systematic study. They objectified multiple hepatic and splenic lesions suggestive of metastases. After an exhaustive study, they concluded that it was a dissemination of a cutaneous malignant melanoma with bilateral choroidal metastases, liver and spleen metastases.

**Figure 3:**
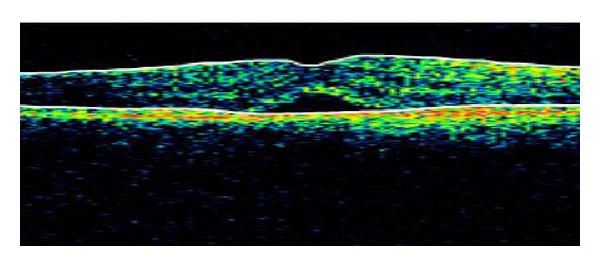
OCT image which shows the improvement of the serous macular detachment in the right eye after palliative chemotherapy.

The patient underwent palliative treatment with chemotherapy with Carboplatin + DTIC and Interleukin + Interferon. The patient experienced with chemotherapy clinical improvement. The visual acuity improved to 20/25 in the RE, with a significant reduction of the macular neurosensory detachment in the RE (Figure 3). Nevertheless the patient died months after the diagnosis of cutaneous melanoma dissemination. Autopsy was performed and histopathological examination of the eye revealed numerous pigmented cells, polygonal in shape with round or oval nuclei. Inmunohistochemistry for S-100, neuron-specific enolase, and HMB-45 antibody showed positive staining reaction of the lesions, so the diagnosis of choroidal metastases from a malignant cutaneous melanoma was confirmed.

## 3. Discussion

Ocular metastases may manifest clinically in different ways, but are sometimes asymptomatic. In our case report, the patient complained of painless reduction of vision and after an exhaustive eye examination, multiple pigmented placoid choroidal lesions located in the posterior pole and midperiphery of both eyes were found. Stephens and Shields reviewed 70 cases of patients with choroidal metastases and found that blurred vision was the reason for consultation in 80% of patients, pain was noted in 14%, photopsia in 13%, red eye and floaters in 7%, and visual fields defects in 3% [2]. Lesions are bilateral in 20–40% of cases and are often located in the posterior pole, probably because blood flow is greater in that area [3].

Differential diagnosis includes primary choroidal melanomas, benign lesions such as hemangiomas and inflammatory granulomas. In our case, due to the clinical presentation, the presence of metastases in other locations and previous history of cutaneous melanoma directed us to the diagnosis of bilateral choroidal metastases in the context of metastatic spread of cutaneous malignant melanoma.

The OCT has revolutionized the field of ophthalmology since its introduction in 1991. In ocular oncology, it gives us high resolution images providing valuable information about the effects that cause tumoral lesions in the retina such as retinal edema, subretinal fluid, retinal atrophy, loss of photoreceptors, and retinal pigment epithelium detachment. OCT aids in the diagnosis, the therapeutic approach and in monitoring response to treatment of tumoral lesions. In our case, it helped us to objectify the subretinal fluid in the macula of the RE and the significant reduction of the fluid after palliative chemotherapy [4].

Dissemination of cutaneous melanoma to the choroid is rare and is a major prognostic factor. Although our patient had had a cutaneous melanoma 6 years ago and considered to be good prognosis by anatomical and pathological features without initial metastases or during the first years of followup, he developed systemic dissemination. Shields et al. in a study of 520 eyes with uveal metastases found that in 2% of cases the primary tumor had its origin in the skin. Most cases of skin tumors that metastasize and with higher mortality rates are from sebaceous cell carcinoma and malignant cutaneous melanoma. In the same study, Shields et al. found that 66% of cases had previous history of primary tumor and patients who had no previous history, the primary tumor was found in 49% [1].

Most recurrences and deaths of patients occur within the first ten years of primary tumor treatment [5]. The prognosis for patients with cutaneous melanoma is related to the depth of dermal invasion. Although melanomas with less than 0.76 mm of thickness are considered to have a 5-year survival rate of 96 to 99%, the reported case developed systemic metastases 6 years after excision. Prognosis when ocular metastases are present is poor with a survival rate less than 8 months in 90% of patients [6].

In general the treatment of ocular metastases is palliative, because the presence of metastases indicates hematogenous spread of tumor. The objectives are therefore to maximize the quality of life and restore or preserve vision. This can be achieved with radiotherapy or chemotherapy or other treatments. In the case reported, the patient experienced clinical improvement of his VA and ocular lesions after treatment with palliative chemotherapy, although the patient died in the following months [6, 7].
